# Impact of Clinical Pharmacist Running Anticoagulation Clinic in Saudi Arabia

**DOI:** 10.3390/jcm12123887

**Published:** 2023-06-07

**Authors:** Abdulrahman Alshaiban, Sirajudeen S. Alavudeen, Ibrahim Alshahrani, Abdulaziz M. Kardam, Ibrahim Mohammed Alhasan, Saleh Abdulrahman Alasiri, Mohammad Tarique Imam, Ziyad Saeed Almalki, Md Sayeed Akhtar

**Affiliations:** 1 King Faisal Medical City, Ministry of Health, Abha 62521, Saudi Arabia; asalshaiban@kfmcity.med.sa (A.A.); i.m.shahrani@kfmcity.med.sa (I.A.); a.kardm@hotmail.com (A.M.K.); i.m.hasan@kfmcity.med.sa (I.M.A.); s.a.asiri@kfmcity.med.sa (S.A.A.); 2 Department of Clinical Pharmacy, College of Pharmacy, King Khalid University, Al-Fara, Abha 62223, Saudi Arabia; sshaik@kku.edu.sa; 3 Department of Clinical Pharmacy, College of Pharmacy, Prince Sattam Bin Abdulaziz University, Al-Kharj 16273, Saudi Arabiaz.almalki@psau.edu.sa (Z.S.A.)

**Keywords:** warfarin, clinical pharmacist, quality of life, anticoagulation, adverse drug reactions

## Abstract

Despite the effectiveness of warfarin in extended anticoagulation, its narrow therapeutic index requires frequent dose adjustments and careful patient monitoring. Thus, we aimed to evaluate the outcomes of clinical pharmacists’ intervention in warfarin therapy management in terms of International Normalized Ratio (INR) control, reduction of bleeding, and hospitalization in a tertiary care hospital. An observational retrospective cohort study was conducted on 96 patients taking warfarin therapy in a clinical pharmacist-led anticoagulation clinic. We observed that 39.6% of patients required dose adjustments at their first and second visits. However, dose adjustments during the third, fourth, and fifth weeks were required at 31.1%, 20.8%, and 4.2%, respectively, to achieve INR levels. We also observed that 36.46% of the patients attained the target INR at baseline, which was increased over the first week to the fifth week to 57.29%, 61.46%, 61.46%, 68.75%, and 85.42%, respectively. No one reported the ADR between the third and fifth weeks. Based on our findings, the study strongly suggests that pharmacists’ interventions can improve the health-related quality of life of patients undergoing warfarin therapy. Thus, competent pharmacy personnel must be a priority in both usual patient care and critical care among primary care networks.

## 1. Introduction

According to the Centers for Disease Control and Prevention (CDC), 18.9% of mortality due to heart diseases has been reported in the Asian population during 2018–2020 among patients who were treated with anticoagulants at some point in patient care [1,2]. Anticoagulants are substances that prevent coagulation and stop the clotting of blood from growing larger in the blood or blood vessels [3]. Warfarin is an age-old anticoagulant that was introduced in the 1950s and became the most commonly used oral anticoagulant. Although many newer anticoagulants have been approved in recent years, warfarin is still considered the drug of choice for long-term or extended anticoagulation in patient care. Warfarin is approved by the United States Food and Drug Administration (USFDA) for the prevention and treatment of venous thromboembolism, prevention of thromboembolic complications in patients with myocardial infarction, atrial fibrillation, and heart valve replacement [4]. Despite its effectiveness, warfarin has a narrow therapeutic index, requiring frequent dose adjustments and careful patient monitoring [5,6]. In many diseased conditions, the effectiveness and safety of warfarin mainly depend on the international normalized ratio (INR) level of the patients. Supratherapeutic levels of warfarin can lead to an increased INR, thereby increasing the risk of bleeding complications. Likewise, subtherapeutic levels will decrease the INR and, consequently, increase the risk of thromboembolic complications. Adverse reactions to anticoagulants have been associated with many factors, such as the complexity of dosing and monitoring, patient compliance, and numerous drug–drug, and drug–food interactions [7,8]. The anticoagulation management service (AMS) has been established to monitor and manage oral and parenteral anticoagulants that decrease the formation of blood clots [9]. Many studies have been conducted to compare the anticoagulation therapy management between conventional primary care physician management and the specialized anticoagulation service rendered by clinical pharmacists in collaboration with physicians. It is evident from the studies that the patients who were managed by specialized anticoagulation services achieved better anticoagulation control in terms of time to achieve and control in the therapeutic range, reducing complication rates by up to 50–90% over those managed by conventional practice [10,11,12,13,14,15]. Moreover, in a survey conducted to evaluate patients’ perceptions of pharmacist involvement with anticoagulation services in a pharmacist-managed anticoagulation clinic, it was found that the majority of patients were comfortable with pharmacists’ services in anticoagulation management, such as monitoring warfarin therapy and its dosage adjustments [16,17]. Hence, it has been proven that the pharmacist’s role in anticoagulation management is multifactorial and can include, but is not limited to, monitoring, dosing, provision of drug information, patient education, drug interaction screening, and research [18]. An evaluation of the anticoagulation service demonstrated that the pharmacist-provided service achieved significantly better INR control as compared to usual care [14]. 

In Prince Faisal Bin Khalid Cardiac Center, Abha, Aseer Province, Saudi Arabia, outpatients on warfarin were generally managed by their family physician. In 2020, a clinical pharmacist-run anticoagulation service was initiated. This is the first of its kind in the southern region of Saudi Arabia. As this anticoagulation clinic is new, there is almost no data available on the clinical benefits of this clinic. Thus, we aimed to evaluate the outcomes of clinical pharmacists’ intervention in the control of warfarin INR level and management of warfarin therapy risk of bleeding and hospitalization in a tertiary care hospital.

## 2. Method

### 2.1. Study Design and Setting

An observational retrospective cohort study was conducted on patients taking warfarin therapy in a clinical pharmacist-led anticoagulation clinic. The study was conducted at the Prince Faisal Bin Khalid Cardiac Center, a tertiary care hospital, in Abha, Aseer Province, Saudi Arabia. 

### 2.2. Models of Care

Usual care model (physician model): Either the treating physician would provide the anticoagulation management, or the cardiologist would care for referred patients in the cardiology clinic. In all cases, the blood samples of the patients are tested for an INR, and the physician would retrieve the results from the computerized laboratory system. The physician would assess the result of the patients and recommend dosage changes if required. Though the plan sounds good, this process was not organized, and all the patients would be scheduled for follow-up on a specific day with no specific time. This procedure made the patients wait for a long time, and the physicians found none-to-minimum time to educate and counsel the patients. Some patients would not return to the physician after getting their anticoagulant drugs from the outpatient pharmacy due to a lack of convenience and ease of physicians’ access [19]. 

Clinical pharmacist-led anticoagulation clinic (ACC): A dedicated space in the cardiology clinic is allotted with access to the hospital information system (HIS) to the clinical pharmacist who is involved in anticoagulation management. A trained, certified clinical pharmacist initiates, refills, and adjusts the dose of oral anticoagulation based on the approved protocol [20]. In addition, the clinical pharmacist reviews the patients’ medical chart, electronic profile, and history and orders laboratory tests as deemed necessary to achieve therapeutic anticoagulation goals. The clinical pharmacist was given flexibility to assess each patient individually to develop patient-specific recommendations. The first visit and the follow-up visits of the patients are scheduled by appointment; there are a few exempted cases for urgent reasons. The patient’s first visit usually lasts approximately half an hour, during which the clinical pharmacist develops a patient-specific care plan. The pharmacist documents all the actions and recommendations in the patient’s chart to be reviewed by the referring physician. During the visit, the clinical pharmacist assesses the patients for any adverse drug reactions, drug–drug or drug–food interactions, safety and effectiveness of therapy, medication knowledge, and patients’ compliance. Pertinent laboratory test values are examined before the initiation and/or adjustment of anticoagulation therapy. As part of patient education, the patients are instructed regarding the indications, adverse effects, and potential complications of anticoagulation therapy. In addition, during the educational session, patients are informed of their role in disease management, the need for compliance with therapy and blood testing, as well as the importance of contacting the anticoagulation clinic with any changes to their health condition and medications. Other factors that might affect anticoagulation therapy, such as diet and alcohol, are also explained. Follow-up visits are approximately 10–15 min long and are scheduled based on patient status and condition, ranging from 3 days for new patients or patients with dosage adjustments to 4–6 weeks for INR-stable patients. 

Documentation of the assessment and then recommendations were made directly into the patient’s chart and remain available to the physicians. The physicians were readily accessible to the pharmacist for discussion of patient-related issues. The clinic protocol outlined mandatory physician contact by the pharmacist for discussion of management (i.e., INR > 5.0, suspicion of serious adverse effects, new clots, or serious bleeding). Patients had the ability to contact either the physician or the pharmacist for anticoagulation-related queries or concerns.

### 2.3. Eligibility Criteria

Inclusion criteria: The selection criteria include adult patients on warfarin therapy for at least the previous year, on regular follow-up in the Anticoagulation Clinic, and having complete follow-up data for the continuous 5 weeks.

Exclusion criteria: Patients who did not need titration of warfarin therapy during their inpatient stay and were discharged with low molecular weight heparin and managed as outpatients. Pregnant women, patients who cannot communicate in Arabic/English, or those who have hearing, or cognitive problems were also excluded from the study. 

### 2.4. Data Extraction

A clinical pharmacist used a data extraction form to extract data from the family physician’s and pharmacist’s written computerized database and electronic records. All the patients who received warfarin therapy during the year 2022 were recruited, and the data were filtered as per the inclusion and exclusion criteria. 

### 2.5. Study Outcome

Patient demographics, indications for warfarin, INR target and duration of therapy/follow-up, the risk for bleeding or thromboembolic events, types of pharmacists’ interventions, and reasons for not achieving target INR were recorded. The primary outcome of this study was INR control, the pattern of INR achievement, and warfarin dose adjustments. The secondary outcomes were hospitalizations due to adverse drug events.

### 2.6. Sample Size Calculation

The sample size was calculated by assuming a population size of 200, a confidence level (95%), and a 5% margin of error. Based on these, 84 patients were required for this study. A total of 300 records were reviewed, and a total of 96 patients were included in the study.

### 2.7. Statistical Analysis

Statistical analysis was performed using Microsoft Excel 2019 (Microsoft Corp., Redmond, WA, USA) and SPSS statistical software for Windows, version 21.0 (SPSS Inc., Chicago, IL, USA). Data are presented in the form of mean ± SD, frequency, and percentage (%), as appropriate. Comparisons between the change in INR for consecutive 5 weeks were conducted by using a one-way ANOVA. Descriptive analysis, such as the Pearson Chi-square test, was applied to observe the differences in rates and proportions. A *p*-value less than 0.05 was considered a significant difference between the groups.

### 2.8. Ethics Approval

The study protocol was approved by the research ethics committee at the King Khalid University (HAPO-06-B-001; dated 22 May 2022), Abha, Saudi Arabia. The study was also approved by the Aseer Institutional Review Board (Registration number: H-06-B-091) in Aseer, Abha, Saudi Arabia. The reported experiment was conducted in accordance with the ethical standards of the institutional and/or national research committee and with the Helsinki Declaration of 1975, as revised in 2008 (https://www.wma.net/wp-content/uploads/2018/07/DoH-Oct2008.pdf; accessed on 5 June 2022).

## 3. Results

A total of 300 records were reviewed, and based on the inclusion and exclusion criteria, we included 96 participants in our study. Table 1 shows the demographic characteristics and related comorbidities among study participants. An almost equal number of male (52.08%) and female (47.92%) participants were recorded in the study.

**Figure 1:**
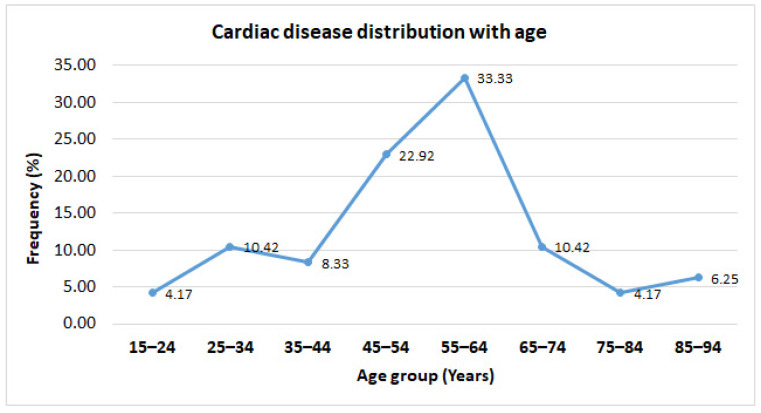
Cardiac disease distribution with age among study participants. % —Percentage.

In our study, we observed that the number of participants was higher in the age groups 45–54 (22.92%) and 55–64 (33.3%) among all the participants. There was a significant difference (*p* < 0.01) between male (91.6%) and female (8.4%) smokers. Most of the patients were suffering from different types of comorbidities, particularly cardiovascular diseases (CVDs) such as valvular disease (58.33%), followed by hypertension with diabetes (22.92%), hypertension (8.34%), and acute coronary syndrome (6.25%). Similarly, Figure 1 indicated that the distribution of cardiac diseases was more prevalent among the age group 45–64 (55.25%).

Table 2 indicates the cause and frequency of warfarin intervention among study participants. Most of the patients using warfarin underwent mitral valve replacement (MVR)-mechanical (33.33%), followed by MVR-bioprosthetic (18.75%), dual aortic valve replacement (AVR), and MVR (16.67%), atrial fibrillation (8.33%), and AVR-mechanical (4.14%). 

**Figure 2:**
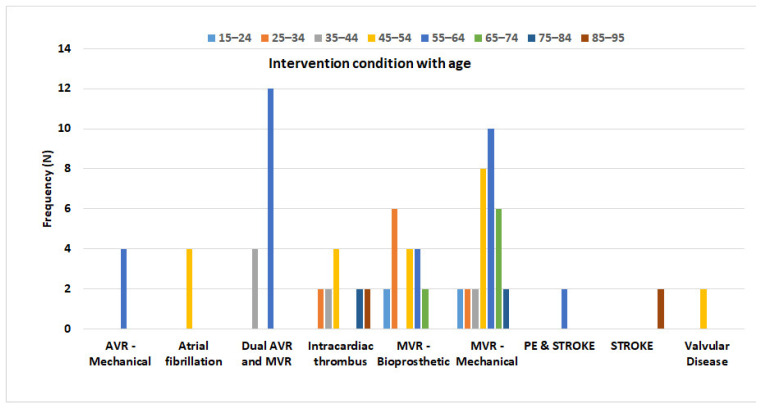
Warfarin intervention with age in different disease indications. N—Number of participants; MVR—Mitral Valve Replacement; AVR—Aortic Valve Replacement; PE—Pulmonary embolism.

Figure 2 depicted the type of interventions in the different categories of age. Importantly, in the age group of 55–64 years, dual MVR and AVR were performed mostly (12.5%) followed by MVR–mechanical (10.41%) and MVR–bioprosthetic and AVR-mechanical (4.16%). However, in the age group of 65–74 years, 6.25% and 2.08% of patients were intervened by MVR–mechanical and MVR–bioprosthetic, respectively.

Table 3 shows the type of clinical pharmacists’ intervention among study participants. Almost 100% of the patients received medication reconciliation and patient counseling during the weekly monitoring of the INR value of warfarin under various disease conditions. Similarly, no drug changes or medication discontinuations were observed during the weekly monitoring of the INR value of warfarin. Around 4.2% of patients were instructed to hold the medication during the first visit and 2.1% of patients during second and fifth visit to a pharmacist-managed anticoagulant clinic. Physicians’ recommendations also declined from the third week (52.1%), followed by the fourth week (20.8%) and the fifth week (14.6%). Around 4.2% of patients reported ADR during the first visit, followed by 2.1% in the second visit. No one reported the ADR between the third and fifth weeks.

Furthermore, Table 3 indicate that the need for dose adjustment dropped every consecutive week. We observed that 39.6% of patients required dose adjustments at their first and second visits. However, dose adjustments during the third, fourth, and fifth weeks were required in 31.1%, 20.8%, and 4.2%, respectively. 

**Figure 3:**
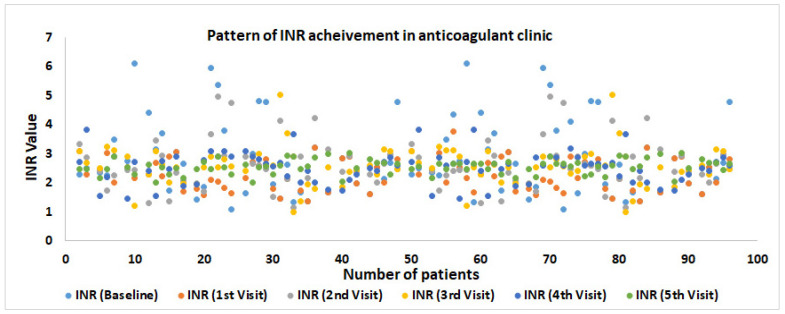
Patterns of INR achievement during anticoagulant therapy among study participants. INR: International normalized ratio; the Green color dot represents all the patients in the target INR range during the fifth week of intervention.

**Figure 4:**
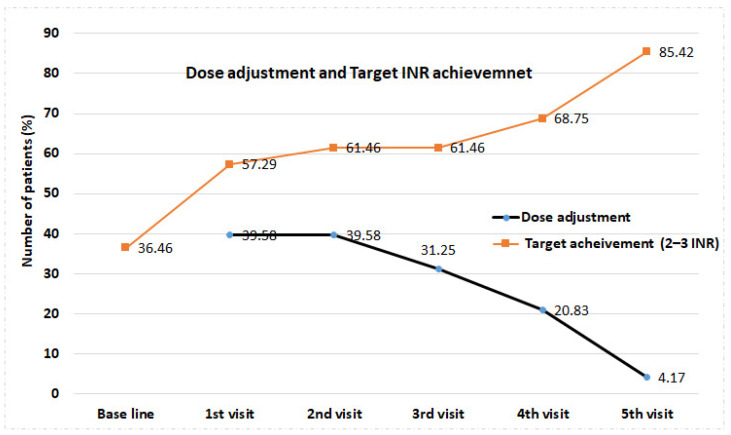
Dose achievement and target INR achievement among study participants. %—Percentage; INR—International normalized ratio.

Table 4 shows the change in weekly dose and INR value post pharmacist intervention among study participants. There was a significant difference (*p* = 0.001) in the weekly dose (in mg) between the baseline and fifth visits among the patients. Concurrently, the dose range also significantly (*p* = 0.001) declined over the baseline (52.5) to the fifth visit (42.5) among the patients. Table 4 and Figure 3 indicate that most of the patients had INR values within the therapeutic range (2–3 INR). We further observed lesser standard deviations over the period from baseline to the fifth visit, which strongly supports the results of Figure 3. We also observed a steep change in target INR achievement among the patients counseled during warfarin therapy by the clinical pharmacist. Only around 36.46% of the patients attained the target INR at baseline, which was increased over the first week to the fifth week to 57.29%, 61.46%, 61.46%, 68.75%, and 85.42%, respectively (Figure 4). 

Table 5 indicates adverse drug reactions and reasons for not achieving the target INR among study participants. Firstly, nose bleeding was reported in 6.3% of patients at base line that decreased to 4.2%, 2.1% and 0.0% in 1st visit, 2nd visit, and 3rd visit respectively. We observed that apart from unknown reasons (41.7%), non-compliance (18.8%) and non-follow-up (16.7%) were the most baseline reason for not achieving the target INR value among the patients. 

However, unknown reasons, non-compliance, and non-follow-up declined to around 0.0% in the fifth visit interestingly. Only 2.1% of cases were reported to receive the subtherapeutic dose at the baseline and third visit. No drug–drug interaction was observed over the period of baseline to the fifth visit. 

## 4. Discussion

Anticoagulation management service (AMS) is needed to improve the quality of patient care and minimize complications as adverse events, particularly due to warfarin therapy [21]. The current study aimed to assess the importance of the clinical pharmacist-led anticoagulation clinic in improving patient-oriented outcomes during the warfarin treatment process. We included an almost similar number of male and female participants in our study based on the study criteria to reduce sex as a confounder. As the previous data recorded, we observed major proportions of patients belonging to the upper middle-aged population (45–64 years) with certain cardiac diseases among those who required warfarin therapy [22,23]. This gives a warning signal to take care of your health and promote a healthy lifestyle, such as regular exercise, a healthy diet, and adhering to the instructions of the healthcare professional for controlling any pre-existing complications, if any. Furthermore, it is highly evident that hypertension, dyslipidemia, diabetes mellitus, and other metabolic disorders are the major modifiable risk factors that contributed to the development of heart valve disease and other cardiovascular diseases (CVD) [24,25]. In line with these findings. We also found that a major number of patients underwent Mitral valve regurgitation (MVR)–mechanical, MVR–bioprosthetic, dual AVR, and MVR, AVR–mechanical than warfarin therapy [26,27,28]. However, more importantly, among the age group of 55–64 years, dual MVR and AVR, MVR–mechanical, and MVR–bioprosthetic and AVR–mechanical were performed in around 27% of patients. This suggests that preventing cardiac disease risk factors will reduce the development of these heart valve disorders [2]. It is well evident that pharmacists play a major role in increasing the patient’s awareness as well as patient compliance in various disease conditions, particularly chronic illness. However, the emerging role of clinical pharmacists in critical care, such as managing the target therapeutic level of narrow safety margin drugs, is also well reported in various studies [29,30]. Consistently, medication reconciliation, patient counseling, and weekly monitoring of INR value were conducted by pharmacists during all five consecutive visits of the patients and impacted the patient-important outcome very effectively [31,32,33]. Pharmacists in our study efficiently managed the ADR, such as nose bleeding, and no one reported this event during the third to fifth visit. We understand that the major reason for managing the ADR was achieving the target therapeutic INR level from supratherapeutic levels, as reported earlier [34,35]. 

Like the previous study, our study further supports the evidence of pharmacist intervention as the need for dose adjustment among the participants was dropped every consecutive week. The decrease in warfarin dose range over the baseline to five consecutive visits to pharmacist-managed anticoagulant clinics is well reported in our study. Furthermore, we also found a steep increase in target INR achievement among the patients counseled during warfarin therapy by the clinical pharmacist, as reported earlier [36,37]. Altogether, both of these results evidence the role of pharmacists in managing warfarin use in critical care, where any medication adventure may be fatal for the patients. This study outcome is consistent with other matched control studies conducted by Falmic et al. in 2021 and many other similar studies [14,32,33,35,38]. In the last segment of our study, we tried to find the reason for not achieving the target INR level of warfarin in our patients. As reported in previous matched control studies, non-follow-up-related decreased patient counseling and non-compliance have emerged as major issues for not achieving the target INR level [14,38,39]. This result supports the above discussion and promotes the importance of clinical pharmacists during warfarin therapy, particularly in the care of cardiac diseases. The Clinical pharmacist model is well accepted by different healthcare providers [40]. However, its implementation involves a few trade-offs, such as increased workload due to lack of knowledge and training and, more importantly, the direct involvement of clinical pharmacists in drug therapy management as a primary care network [41,42]. In corroboration of the above discussion, our study strongly emphasizes the role of pharmacists in the safe and effective use of warfarin in both usual patent care and critical care. Moreover, through the primary care network, the transition to more direct patient care by clinical pharmacists from transactional care is the future trend. Thus, further data regarding the implementation of the clinical pharmacist-led model will be required to open the way and can be considered in future research. 

## 5. Limitations

Due to the nature of a retrospective cohort study, the number of confounders was not controlled, and neither the duration of counseling nor the content of counseling were recorded. Although we found a very clear idea about the benefits of pharmacists’ involvement in patient care, the lack of matched control is another major limitation of this study. Future research with an approach with more study samples and evidence for protocol-driven care would help get more insight into the study context.

## 6. Conclusions

Based on our findings and discussion, we suggest that the intervention of clinical pharmacists during warfarin therapy may improve the target INR control. This not only reduced the ADRs like bleeding but also reduced further complications due to warfarin-related benefits in any specific circumstances, such as cardiac care. Pharmacists’ interventions can improve the health-related quality of life of patients undergoing warfarin therapy. Thus, competent pharmacy personnel must be a priority in both usual patient care and critical care among primary care networks.

## Figures and Tables

**Table 1:** Demographic characteristics and related comorbidity among study participants.

**Gender**	**Male N (%)**	**Female N (%)**	***p*-Value**
	50 (52.08)	46 (47.92)	*p* = 0.683
**Age Group (Years)**	**Male N (%)**	**Female N (%)**	***p*-Value**
15–24	4 (8)	0 (0)	---------
25–34	6 (12)	4 (8.7)	*p* = 0.527
35–44	4 (8)	4 (8.7)	*p* = 1.000
45–54	16 (32)	6 (13.04)	*p* = 0.033
55–64	10 (20)	22 (47.83)	*p* = 0.034
65–74	2 (4)	8 (17.39)	*p* = 0.058
75–84	2 (4)	2 (4.35)	*p* = 1.000
85–94	6 (12)	0 (0)	---------
Total	50 (52.08)	46 (47.92)	
**Smoking Habits**	**Male N (%)**	**Female N (%)**	***p*-Value**
	88 (91.6)	8 (8.4)	*p* = 0.000
**Type of Diseases**	**Male N (%)**	**Female N (%)**	***p*-Value**
AF	4 (8)	0 (0)	---------
HTN	4 (8)	4 (8.7)	*p* = 1.000
VD	28 (56)	28 (60.87)	*p* = 1.000
ACS	4 (8)	2 (4.35)	*p* = 0.414
HTN + Diabetes	10 (20)	12 (26.09)	*p* = 0.670
Total	50 (100)	46 (100)	

N—Number of participants; %—Percentage; AF—Atrial fibrillation; HTN—Hypertension; ACS—Acute coronary syndrome; VD—valvular disease. *p*-value less than 0.05 is considered as a difference between the groups. *p*-values obtained by Chi-squared test for categorical variables.

**Table 2:** Cause and frequency for warfarin intervention among study participants.

Indications	N (%)	*p*-Value
AVR–Mechanical	4 (4.17)	*p* = 0.000
Atrial fibrillation	8 (8.33)	
Dual AVR and MVR	16 (16.67)	
Intracardiac thrombus	12 (12.5)	
MVR–Bioprosthetic	18 (18.75)	
MVR–Mechanical	32 (33.33)	
PE and Stroke	2 (2.02)	
Stroke	2 (2.02)	
Valvular Disease	2 (2.02)	
Total	96 (100)	

N—Number of participants; %—Percentage; MVR—Mitral Valve Replacement; AVR—Aortic Valve Replacement; PE—Pulmonary embolism. *p*-value less than 0.05 is considered as a difference between the groups. *p*-values obtained by Chi-squared test for categorical variables.

**Table 3:** Type of clinical pharmacist’s intervention among study participants.

Type of Clinical Pharmacist’s Intervention	1st Visit		2nd Visit		3rd Visit		4th Visit		5th Visit		*p*-Value
	N	%	N	%	N	%	N	%	N	%	
Medication reconciliation (Yes)	96	100	96	100	96	100	96	100	96	100	
Medication discontinuation (Yes)	0	0.0	0	0.0	0	0.0	0	0.0	0	0.0	
Hold the medication (Yes)	4	4.2	2	2.1	0	0.0	0	0.0	2	2.1	
Drug addition (Yes)	0	0.0	0	0.0	2	2.1	0	0.0	0	0.0	
Drug change (Yes)	0	0.0	0	0.0	0	0.0	0	0.0	0	0.0	
Dose adjustment (Yes)	38	39.6	38	39.6	30	31.2	20	20.8	4	4.2	*p* = 0.000
ADR reporting (Yes)	4	4.2	2	2.1	0	0.0	0	0.0	0	0.0	
Patient counseling (Yes)	96	100	96	100	96	100	96	100	96	100	
Physicians’ recommendation (Yes)	50	52.1	50	52.1	50	52.1	20	20.8	14	14.6	

N—Number of participants; %—Percentage; *p*-value less than 0.05 is considered as a difference between the groups. *p*-values obtained by Chi-squared test for categorical variables.

**Table 4:** Change in the weekly dose and INR value post pharmacist intervention among study participants.

Change in Weekly Dose							
Drug	Baseline	1st Visit	2nd Visit	3rd Visit	4th Visit	5th Visit	*p*-Value
Weekly Dose (mg) (Mean ± SD)	28.99 ± 11.81	27.74 ± 12.28	28.33 ± 11.45	29.48 ± 12.05	30.70 ± 12.58	31.60 ± 13.35 *	0.001
Dose Range (Min–Max)	52.50 (3.50–56)	47 (8–55)	47 (9–56)	43 (10–56)	43 (12–55)	42.5 (12–54.5)	0.001
Change in Weekly INR							
INR Value	Baseline	1st Visit	2nd Visit	3rd Visit	4th Visit	5th Visit	*p*-Value
INR (Mean ± SD)	2.87 ± 1.31	2.5 ± 0.95 *	2.63 ± 0.82 *	2.62 ± 0.79 *	2.58 ± 0.58 *	2.63 ± 0.47 *	0.001
INR Range (Min–Max)	5.02 (1.10–6.12)	5.32 (1.38–6.70)	3.8 (1.1604.96)	4.55 (1.01–5.56)	2.64 (1.47–3.91)	0.4 (1.64–4.26)	0.001

INR—International normalized ratio; Min—Minimum; Max—Maximum. *p*-value less than 0.05 is considered as a difference between the groups. All the changes in values of weekly dose and INR range are compared with baseline. * *p* = 0.001 vs. Baseline.

**Table 5:** Adverse drug reactions and reasons of not achieving target INR among study participants.

	Number of Patients					
Adverse Drug Reactions	Baseline N (%)	1st Visit N (%)	2nd Visit N (%)	3rd Visit N (%)	4th Visit N (%)	5th Visit N (%)
Nose bleeding	6 (6.3)	4 (4.2)	2 (2.1)	0 (0.0)	0 (0.0)	0 (0.0)
Reasons of Not Achieving Target INR	Baseline N (%)	1st Visit N (%)	2nd Visit N (%)	3rd Visit N (%)	4th Visit N (%)	5th Visit N (%)
Non-compliance	18 (18.8)	24 (25)	16 (16.7)	8 (8.3)	4 (4.2)	2 (2.1)
Dose hypersensitivity	2 (2.1)	0 (0.0)	0 (0.0)	0 (0.0)	2 (2.1)	0 (0.0)
Sub-therapeutic dose	2 (2.1)	0 (0.0)	0 (0.0)	2 (2.1)	0 (0.0)	0 (0.0)
No follow-up	16 (16.7)	6 (6.3)	6 (6.3)	0 (0.0)	0 (0.0)	0 (0.0)
Drug–Drug interaction	0 (0.0)	0 (0.0)	4 (4.2)	0 (0.0)	0 (0.0)	0 (0.0)
Unknown reason	40 (41.7)	12 (12.5)	12 (12.5)	18 (18.8)	2 (2.1)	0 (0.0)

N—Number of participants; %—Percentage; INR—International normalized ratio.

## Data Availability

The data presented in this study are available on request from the corresponding author. The data are not publicly available due to data confidentiality.
